# Associations of breastfeeding duration with serum lipid values from infancy until age 20 years – the STRIP study

**DOI:** 10.1177/14034948231183030

**Published:** 2023-06-30

**Authors:** Tomi T. Laitinen, Emma Saha, Katja Pahkala, Noora Kartiosuo, Joel Nuotio, Hanna Lagström, Jorma Viikari, Tapani Rönnemaa, Antti Jula, Olli Raitakari, Harri Niinikoski

**Affiliations:** 1 Research Centre of Applied and Preventive Cardiovascular Medicine; University of Turku, Turku, Finland; Centre for Population Health Research, University of Turku and Turku University Hospital, Turku, Finland; 2 Paavo Nurmi Centre, Unit for Health and Physical Activity, University of Turku, Turku, Finland; 3 Heart Center, Turku University Hospital and University of Turku, Turku, Finland; 4 Department of Clinical Medicine, University of Turku, Finland; 5 Department of Medicine, University of Turku, Turku, Finland; 6 Department of Medicine, University of Turku, Finland; 7 Department of Clinical Physiology and Nuclear Medicine, Turku University Hospital, Turku, Finland; 8 Department of Pediatrics, University of Turku, Turku, Finland; 9 Department of Pediatric and Adolescent Medicine, Turku University Hospital, Turku, Finland

**Keywords:** Breastfeeding, childhood, cholesterol, lipids

## Abstract

**Background and aims:**

The effect of breastfeeding duration on childhood lipid levels has remained controversial. In this study, we aimed to establish the long-term associations of breastfeeding duration with future levels of total cholesterol, high-density lipoprotein (HDL) cholesterol, non-HDL cholesterol and low-density lipoprotein cholesterol. In addition, we report lipid levels at the age of seven months depending on the child receiving any breastmilk.

**Methods:**

The sample comprised 999 children participating in the prospective Special Turku Coronary Risk Factor Intervention Project (STRIP). Serum lipid profile was studied at the ages of seven months and 13 months, and annually thereafter until the age of 20 years. Duration of breastfeeding was inquired, and infants were divided into those who received or did not receive any breast milk at the age of seven months (*n*=533 and *n*=466, respectively). In addition, breastfeeding duration groups (any breastfeeding for 0–4 months, 4–6 months, 6–9 months, and >9 months) were formed.

**Results:**

At the age of seven months infants who at that time received breast milk had higher serum HDL cholesterol (0.95±0.21mmol/l *vs*. 0.90±0.19 mmol/l; *p*=0.0018), non-HDL cholesterol (3.38±0.78 mmol/l *vs*. 3.01±0.67 mmol/l; *p*<0.001) and total cholesterol levels (4.33±0.80 mmol/l *vs*. 3.91±0.69 mmol/l; *p*<0.001) than their peers who did not receive breast milk. From two to 20 years of age serum lipid levels showed no consistent differences between the breastfeeding duration groups.

**Conclusions:**

**Our long-term data showed that duration of breastfeeding has no consistent associations with serum lipid concentrations in healthy individuals aged two to 20 years.**

**Clinical Trial Registration:**

www.clinicaltrials.gov, unique identifier NCT00223600.

## Introduction

It has been previously shown that living conditions, growth and nutrition in foetal life and early childhood influence the risk of chronic non-communicable diseases, for example, cardiovascular diseases, later in adulthood [1,2]. Breast milk is considered optimal nutrition for infants and therefore the World Health Organization recommends exclusive breastfeeding for six months and partial breastfeeding up to two years of age [3]. Human breast milk is high in energy and rich in lipids, especially triglycerides, and cholesterol, while in infant formula milks cholesterol contents are lower [4,5]. Therefore, breast-fed infants show substantially higher serum cholesterol concentration compared with formula-fed ones during the first months of life [6,7].

Breastfeeding obviously has many short-term and long-term benefits on the child as well as the mother [8]. Breastfeeding might, for example, reduce the risk of overweight [9] and diabetes [10] later in life. There have also been findings that breast-fed men have better brachial endothelial function in adulthood [11]. While high serum total and low-density lipoprotein (LDL) cholesterol concentrations are major risk factors for atherosclerotic cardiovascular disease, the effects of breastfeeding duration on weight status and serum cholesterol values in adulthood have remained controversial. For example, Owen et al. [12,13] and Singhal et al. [14] have reported that infants who received breast milk had lower cholesterol levels in adulthood than formula-fed infants, whereas Rudnicka et al. [15], Pirilä et al. [16] and Huttunen et al. [17] have found no relation between breastfeeding and cholesterol levels later in life. It is of interest that maternal diet and weight status influence breast milk fatty acid composition [18].

The STRIP study (Special Turku coronary Risk factor Intervention Project) is a randomized long-term intervention trial where detailed coronary heart disease risk factors, including serum cholesterol values and anthropometrics, have been regularly measured from age seven months to age 20 years in a total of 1062 healthy subjects [7,19]. Our aim here is to establish the long-term effects of breastfeeding duration on future serum cholesterol concentrations. This is especially important since the roots of atherosclerotic cardiovascular diseases are known to expand into infancy and breastfeeding may be a part of the process known as programming.

## Materials and methods

### Study design and study subjects

The STRIP study is an infancy-onset prospective, randomized, controlled trial which aims to prevent atherosclerosis risk factors in healthy children and young adults [7,19,20]. In the city of Turku, Finland, families of six-month-old infants were recruited from well-baby clinics between February 1990 and June 1992, and, subsequently, altogether 1062 infants (56.5% of the eligible age cohort) were randomly allocated to an intervention group (*n*=540) or to a control (*n*=522) group at the age of seven months.

The intervention group received individualized low dietary saturated fat and low cholesterol counselling at least biannually until 20 years of age [7,19]. The main goal of the dietary counselling has been the replacement of saturated fat with unsaturated fat in the child’s diet. Until the age of seven years, counselling was given to the parents and thereafter progressively more information was given directly to the child. The dietary recommendations were based on Nordic Nutrition Recommendations [21]. The control group was seen biannually until the child was seven years old and from then on annually until the age of 20 years. The control group did not receive any specific intervention focused on the prevention of atherosclerosis risk factors, only the basic health education was given. Both study groups met the same study personnel and similar measurements were done, all in one study centre.

The study was approved by the Joint Commission on Ethics of the Turku University and the Turku University Central Hospital. The study was carried out in accordance with The Code of Ethics of the World Medical Association (Declaration of Helsinki) for experiments involving humans. Written informed consent was received from the parents at the beginning of the study and from the children at 15 and 18 years of age.

### Laboratory methods

A non-fasting venous blood sample was taken at the ages of seven months, 13 months and annually from ages two to four years. Fasting blood samples were drawn at the age of five years and annually thereafter (excluding ages six and eight years). After the serum has been clotted at room temperature and centrifugated at low speed (at 3400 *g* for 12 min) it was separated and stored at −25°C for maximally two weeks and thereafter at −75°C. Serum total cholesterol concentration was analysed with a fully enzymatic cholesterol oxidase p-aminophenazone method (Merck, Darmstadt, Germany). Serum high-density lipoprotein (HDL) cholesterol concentration was determined after precipitation of the Apo B-containing lipoprotein particles with dextran sulphate 500,000 [22]. Triglyceride concentrations were measured from the fasting blood samples with the colorimetric glyserol-3-phosphate oxidase p-aminophenazone method (Merck) from age five years onwards. LDL cholesterol concentration was calculated from the Friedewald formula [23]. All children and adolescents had serum triglyceride concentration <4.52 mmol/l. Non-HDL cholesterol concentration was calculated as non-HDL cholesterol = total cholesterol – HDL cholesterol.

### Breastfeeding

At the first study visits the mothers were asked what kind of milk baby was currently used and how long the infants had been breastfed (*n*=999). All mothers were encouraged to breastfeed for as long as they felt comfortable and breast milk or infant formula was suggested to continue until the age of 12 months. Solid foods were increasingly given to all infants according to recommendations in years 1990–1992, that is, from the age of 3–5 months onwards.

In statistical analyses we categorized subjects into breastfeeding duration groups according to *partial* breastfeeding duration, that is, having any breast milk was considered decisive. Consequently, four groups were formed based on the duration of *any* breastfeeding duration 0–4 months (*n*=287), 4–6 months (*n*=160), 6–9 months (*n*=256) and >9 months (*n*=297). These groups were formed because of the current infant feeding recommendations: exclusive breastfeeding is recommended until six months and solid foods are started not earlier than four months but not later than six months of age. Further, to analyse the ‘acute’ effects of breast milk on cholesterol values *during* breastfeeding, infants were divided into those who still received any breast milk (i.e. received at least some breast milk) at the age of seven months (*n*=533*)* and those who were weaned before the age of seven months (*n*=466).

### Statistical analyses

Duration of breastfeeding was used as a categorical variable in study with the four above-mentioned categories and also as a continuous variable (months of breastfeeding). Cochran–Mantel–Haenszel test was used to study STRIP intervention–control group differences in duration of breastfeeding, and since there was non-association (*p*>0.05) the intervention and control groups were analysed combined. Differences between sexes in the duration of breastfeeding were also tested with Cochran–Mantel–Haenszel test. Repeated measures analysis of covariance model was used to study the longitudinal differences between the breastfeeding groups in total cholesterol, HDL cholesterol, HDL cholesterol/total cholesterol ratio, LDL cholesterol, non-HDL cholesterol and triglycerides beginning at age two years. The age of two years was chosen as the youngest age for the analyses because at two years no child received any breast milk. Serum triglyceride values were log_e_-transformed for the analyses. For multiple comparisons of breastfeeding groups, Tukey–Kramer-corrected *p*-values were calculated. The repeated measures linear regression analyses were repeated using the continuous breastfeeding variable. All models included age and sex as covariates. An interaction term for the breastfeeding duration and age was included in separate analyses. The analyses revealed no significant age-interactions, indicating that the associations of the duration of breastfeeding with the serum lipids were similar irrespective of age. All statistical analyses were done with the statistical software package SAS release 9.4 (SAS Institute Inc., Cary, NC, USA). *p*-values <0.05 were considered significant.

## Results

Breastfeeding continued for 7.2±3.8 months in girls and 6.7±3.9 months in boys. There were no differences in the proportions of boys and girls (*p*=0.08) nor in the proportions of intervention and control children (*p*=0.15) in the four breastfeeding duration groups. Twenty-six per cent of the girls and 31.4% of the boys (28.7% of the intervention children and 28.6% of controls) received breast milk for less than four months, 14.2% of girls and 17.8% of boys (12.7% of intervention children, 19.6% of controls) received breast milk for 4–6 months, 27.4% of girls and 23.8% of boys (26.9% of intervention children, 24.3% of controls) received breast milk for 6–9 months, and 30.2% of girls and 29.2% of boys (31.8% of intervention children, 27.5% of controls) received breast milk for > 9 months.

**Figure 1.**
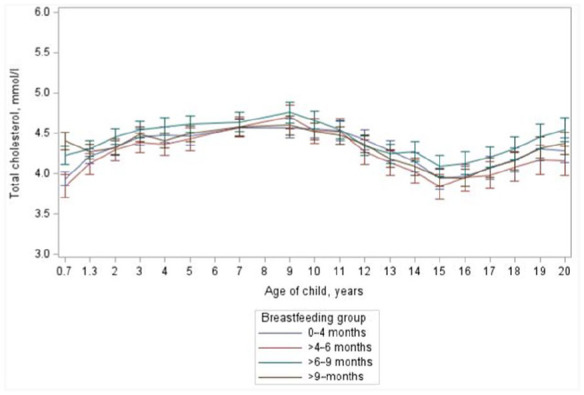
Mean total cholesterol concentrations of children with different duration of breastfeeding from the age of seven months to the age of 20 years. Statistical analyses performed between ages two years and 20 years between feeding groups *p*=0.046. Continuous model *p*=0.86.

At the age of seven months infants who still received breast milk had higher serum total cholesterol values (4.33±0.80 mmol/l) than formula-fed children (3.91±0.69 mmol/l; *p*<0.001) but the effect disappeared soon after weaning. From two to 20 years of age we found no consistent dose–response relationship between the duration of breastfeeding and subsequent serum total cholesterol values (Figure 1). In the analyses, the duration of breastfeeding showed a significant main effect on total cholesterol concentration (*p*=0.046; Figure 1), but when pair-wise analyses comparing the different breastfeeding groups were formed, a tendency towards significant difference was found only between the children breastfed for 4–6 months and 6–9 months (*p*=0.065). Moreover, when duration of breastfeeding was used as a continuous variable (Table I), no association with subsequent total cholesterol values was found (*p*=0.86, Table I). At the age of 20 years, total cholesterol levels did not differ (*p*=0.40) between different breastfeeding duration groups.

**Table I.** Associations between breastfeeding duration (continuous variable, in months) and serum cholesterol values.

	Estimate	SE	*p*
Total cholesterol (mmol/l)	**−0.00092**	**0.005156**	**0.86**
Non-HDL cholesterol (mmol/l)	0.000325	0.004881	0.9**5**
HDL cholesterol (mmol/l)	**−**0.0013	0.001695	0.44
HDL/total cholesterol ratio	**−**0.00025	0.000426	0.55
LDL cholesterol (mmol/l)	0.001448	0.005117	0.78

Repeated mixed model; adjusted for age and sex.

The estimate indicates change in the outcome variable when breastfeeding duration increases by one month.

SE: standard error; HDL: high-density lipoprotein; LDL: low-density lipoprotein.

**Figure 2.**
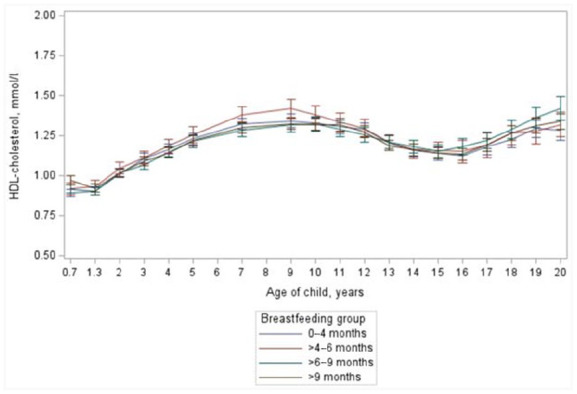
Mean serum high-density lipoprotein (HDL) cholesterol concentrations of children with different duration of breastfeeding from the age of seven months to the age of 20 years. From age two years to 20 years between feeding groups *p*=0.37. Continuous model *p*=0.44.

Similar to total cholesterol concentration, serum HDL cholesterol levels were higher at the age of seven months in those who were still receiving breast milk (0.95±0.21 mmol/l *vs*. 0.90±0.19 mmol/l (*p*=0.0018)) but there was no difference between the breastfeeding groups at later age from two to 20 years of age (*p*=0.37; Figure 2). Similar results were found when breastfeeding was used as a continuous variable (*p*=0.44). At 20 years there were no differences in HDL cholesterol between breastfeeding duration groups (*p*=0.55). The association of breastfeeding with HDL cholesterol concentrations was different between sexes (breastfeeding × sex-interaction *p* < 0.05), thus the girls and the boys were further analysed separately. In both sexes, however, the duration of breastfeeding variable showed no significant main effect on HDL cholesterol concentration.

The ratios of serum HDL cholesterol concentration to total cholesterol concentration also differed at the age of seven months and was higher in infants receiving breast milk (0.24±0.06) than in their formula-fed peers (0.22±0.06) (*p*=0.009). We found no differences in HDL cholesterol/total cholesterol ratios between girls with different duration of breastfeeding (*p*=0.52) but, instead, in boys the ratio differed markedly (*p*=0.002), being higher (0.30) in those who had had breast milk for 4–6 months than in those who had had breast milk for 6–9 months (0.27). However, there was no linear effect between longer duration of breastfeeding and HDL cholesterol/total cholesterol ratios and, when breastfeeding was analysed as a continuous variable, no effect on HDL cholesterol/total cholesterol ratios was found (*p*=0.55; Table I).

**Figure 3.**
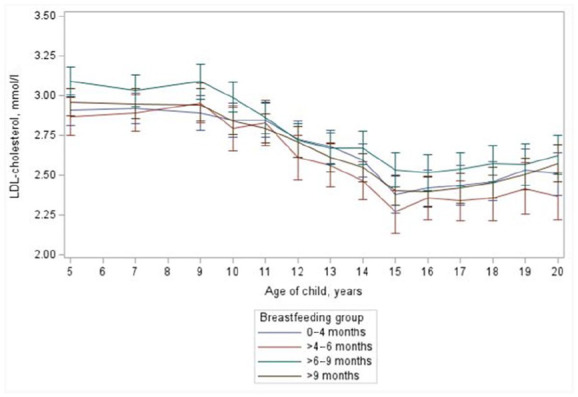
Mean serum low-density lipoprotein (LDL) cholesterol concentrations of children with different duration of breastfeeding between ages 5 years and 20 years. From age five years to 20 years between feeding groups *p*=0.13. Continuous model *p*=0.78

Serum LDL cholesterol (*p*=0.13; Figure 3) and triglyceride (*p*=0.66) concentrations showed no difference from five to 20 years of age between children with different duration of breastfeeding. Because the blood samples were non-fasted before five years of age, we also analysed the association of duration of breastfeeding with non-HDL cholesterol concentrations. At the age of seven months, breast-fed children had higher serum non-HDL cholesterol than formula fed (3.38±0.78 mmol/l *vs*. 3.01 ± 0.67 mmol/l (*p*<0.001). Similar to the association with total cholesterol, the duration of breastfeeding variable showed in the longitudinal analyses a significant main effect on non-HDL cholesterol concentration (*p*=0.046) but there was no consistent dose–response relationship between them. In the pair-wise analyses, a significant difference was found only between those breastfed for 4–6 months and those who had been breast fed for 6–9 months (*p*=0.019). When duration of breastfeeding was used as a continuous variable, no association with non-HDL cholesterol concentration was found (Table I; *p*=0.95). At the age of 20 years there were no differences in non-HDL cholesterol (*p*=0.52) or LDL cholesterol (*p*=0.28) concentrations between groups with different duration of breastfeeding.

## Discussion

In this longitudinal study extending across the first 20 years of life, we did not find evidence to indicate that breastfeeding duration was associated with serum cholesterol concentrations in a dose–response manner from early childhood to adulthood. Participants who had been breastfed for nine months or longer had similar cholesterol concentrations at the age of 20 years as those who had been breastfed for less than four months. These data are unique since there have been no previous studies that would have enabled the investigation of associations of breastfeeding duration with serum lipids assessed up to 19 times beginning from infancy until early adulthood.

Previous studies have shown that serum cholesterol concentrations were higher in breastfed infants than in formula-fed infants during the breastfeeding [6,7,24]. Accordingly, we found higher total cholesterol values in breastfed infants than their formula-fed peers at the age of seven months, the difference being approximately 0.4 mmol/l, that is, 10%. Also, serum HDL cholesterol and non-HDL cholesterol were higher at the age of seven months in those who still received breast milk compared with those who were already weaned, indicating that breast milk rises all cholesterol values during the period of breastfeeding. Previous reports have shown that breastfed infants have significantly higher LDL cholesterol levels than formula-fed infants [6,24,25], which is in line with our finding of breastfeeding effects on non-HDL cholesterol levels. Kallio et al. [6] found that serum LDL cholesterol concentrations indeed explain most of the difference in total cholesterol levels between breastfed and weaned infants. Also, in our study the difference was larger in non-HDL than in HDL cholesterol values between breastfed and formula-fed children.

Even though serum cholesterol levels are higher during breastfeeding than formula feeding this effect seems to disappear soon after weaning. A large meta-analysis of 46 cholesterol studies found no associations between duration of breastfeeding and serum cholesterol values in adults ⩾20 years of age [26]. Our results support these findings as we did not find any dose–response associations between duration of breastfeeding and serum cholesterol values from two to 20 years of age.

We observed a statistically significant difference in serum non-HDL cholesterol concentration and a borderline significant difference in total cholesterol concentration from two to 20 years of age between those who had been breastfed for 4–6 months compared with their peers breastfed for 6–9 months. Interestingly, total cholesterol values of the children who were breastfed either for longer than nine months or less than four months fell between the values of those groups, indicating that there must be some factors other than breastfeeding mediating the difference. A meta-analysis [13] has shown by that breastfeeding did not have any effect on total cholesterol or LDL cholesterol values between ages 13 and 16 years, but in adulthood (>17 years of age) serum total cholesterol and LDL cholesterol concentrations tended to be lower among breastfed than formula-fed subjects.

Considering HDL cholesterol, in our study the influence of breastfeeding on HDL cholesterol was close to significant in both sexes (*p*=0.056 in girls and *p*=0.07 in boys). In pair-wise analyses, in girls the difference was biggest with groups of breastfeeding for 6–9 months and > 9 months (*p*=0.055): those who were breastfed for 6–9 months had higher HDL cholesterol levels than those breastfed for a longer time. In boys, the tendency was seen only between groups of breastfeeding for 4–6 months and 6–9 months (*p*=0.059). These findings indicate no dose response between breastfeeding duration and future HDL cholesterol values in girls or boys in our study. Consistently with these results, Martin et al. found no correlation between breastfeeding and apolipoprotein A1, which is the main protein constituent of HDL particles, at the age of 11.5 years [27]. In addition, Wong et al. [28] showed that breastfeeding duration associated with lower cardiometabolic risk score but not in HDL cholesterol concentrations at 3–6 years of age.

Major strengths of the study are the uniquely long 20-year follow-up period beginning in infancy, a large number of participants and use of well-established laboratory methods. The breastfeeding data were collected prospectively only from the first study visit at the age of seven months, which can be considered a limitation. In addition, we could not examine the effects of full breastfeeding in this study. Further, data on the duration of breastfeeding was collected retrospectively in those infants who had been breastfed for less than seven months. We also acknowledge that because half of the study participants had received dietary intervention affecting serum lipids, the lipid levels in this cohort likely are lower compared with a general population of same aged peers. Moreover, although the control group children did not receive dietary counselling, they were probably more aware of their health-related factors than typical Finnish children. The control families completed food records similar to the intervention peers and received their serum cholesterol values, which could have inadvertently caused them to modify their behaviour and diet, and subsequently serum lipids. Overall, a potential limitation of the STRIP trial is for selection bias in the initial recruitment of the participants, in which families who took part in the trial might have been more interested in health issues. Further, approximately half of the study subjects discontinued the trial before the 20-year visit at the end of the intervention period. However, there have been no differences in baseline variables such as serum lipids between those who continued and those who were lost from the follow-up in the study [19].

In conclusion, our exceptionally long-term data showed that duration of breastfeeding has no consistent association with serum cholesterol concentrations in healthy individuals between ages two and 20 years.
